# Magnetic resonance imaging appearances of the capsulo-osseous layer of the iliotibial band and femoral attachments of the iliotibial band in the normal and pivot-shift ACL injured knee

**DOI:** 10.1007/s00256-018-3128-9

**Published:** 2018-12-28

**Authors:** Monica Khanna, Chimnay Gupte, Alexander Dodds, Andy Williams, Miny Walker

**Affiliations:** 1 Department of Clinical Imaging, Imperial College Healthcare NHS Trust, Praed Street, London, W2 1NY UK; 2 Cheltenham and Gloucester Hospitals, Cheltenham, UK; 3 Fortius Clinic, Fitzhardinghe Street, London, W1H 6EQ UK

**Keywords:** Capsulo-osseous layer, Femoral attachments, Anterolateral rotatory instability, ACL, Pivot shift, MRI

## Abstract

**Background:**

Biomechanical evidence suggests that the anterolateral structures of the knee may be important restraints against anterolateral rotatory instability (ALRI) in the setting of anterior cruciate ligament (ACL) injury.

**Objective:**

To describe the anatomy and presence of injury of the capsule-osseous layer of the iliotibial band (CITB), the iliotibial band, and its deep distal femoral attachments in patients with a ‘normal’ knee (no pivot-shift bone marrow edema (BME) pattern) and patients with a pivot-shift BME pattern indicative of a pivot-shift injury associated with ACL tears.

**Methods:**

Group 1: 20 consecutive patients with no MRI evidence of pivot-shift injury and group 2: 20 consecutive patients with a pivot-shift BME pattern on MRI were identified. Retrospective consensus analysis of the anatomy and appearances of the CITB and the ‘proximal’ and ‘epicondylar’ distal femoral attachments of the ITB was performed for each MRI by two experienced musculoskeletal radiologists.

**Results:**

The positive predictive value (PPV) of CITB injury for pivot-shift ACL injury was 74%, negative predicted Value (NPV) was 80%. The PPV for injury of the ‘proximal’ ITB femoral attachment with pivot-shift ACL injury was 93%, NPV was 84%. The PPV for ‘epicondylar’ iliotibial femoral attachment injury was 62%, NPV was 45%.

**Conclusions:**

Injury of the CITB and ‘proximal’ deep femoral attachments of the ITB are good markers for ACL injury even in the absence of a Segond fracture and should be evaluated on all MRIs as they may prove important in the further management of ALRI.

## Introduction

**Fig. 1:**
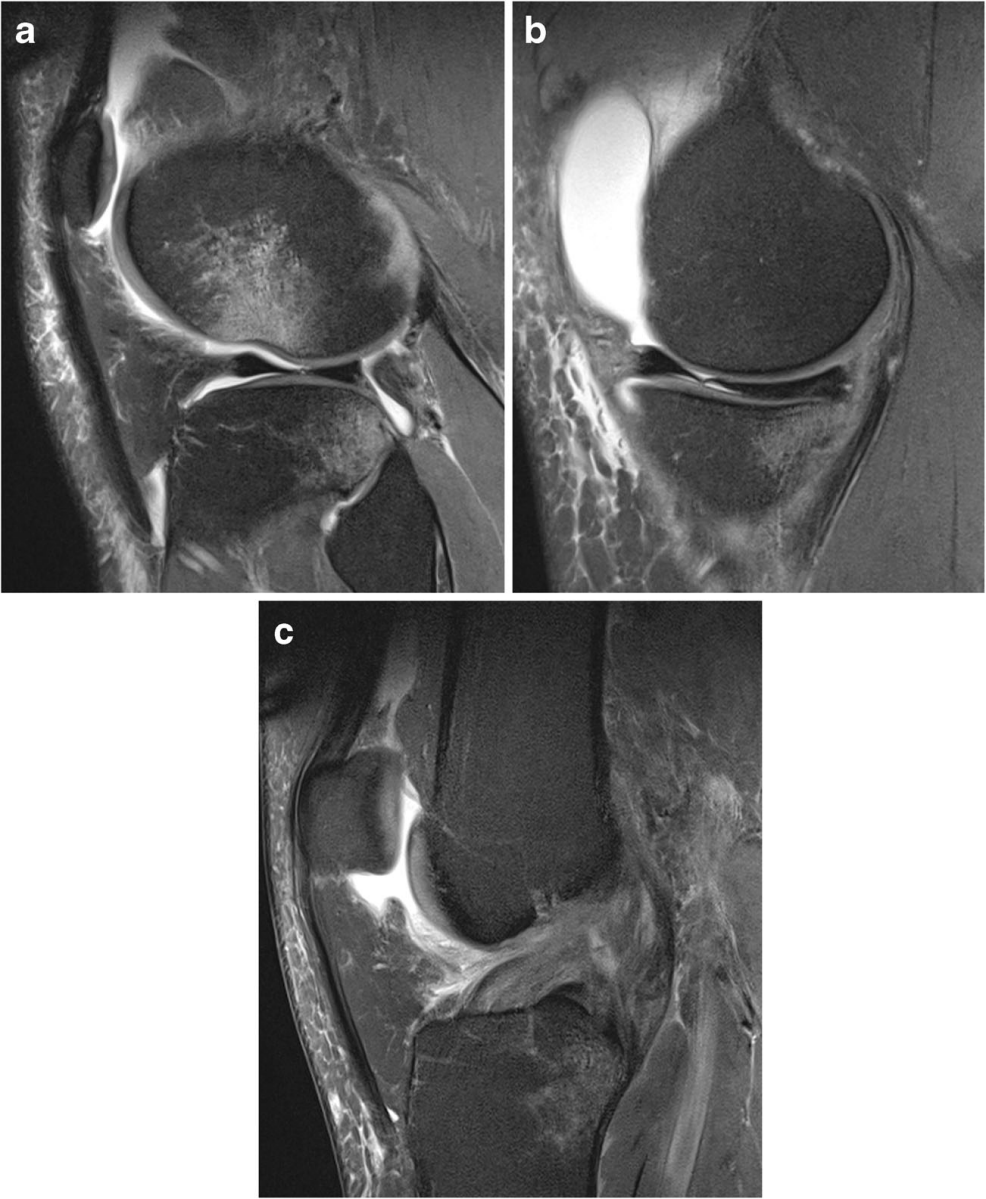
**a** Sagittal proton density fat-saturated (PD FS) image demonstrating subarticular bone marrow edema (BMO) within the lateral sulcus terminalis and posterolateral tibial plateau and **b** posteromedial tibial plateau consistent with a pivot-shift injury. Sagittal PD FS image (**c**) demonstrating an ACL tear in a pivot-shift injured knee

Pivot-shift BME pattern on MRI is a well-recognized indicator of ACL injury in the acutely injured knee. It is increasingly recognized that associated injuries of the anterolateral structures in pivot-shift injury are important (Fig. 1a-c).

Damage to the anterolateral structures of the knee, in particular the part that attaches to the Segond fracture fragment, has been a recent focus of research. Segond first described it in 1879 as a “pearly resistant fibrous band” at the site of the Segond fracture at the anterolateral tibial plateau [1].

Subsequently, its anatomy, function, and biomechanical importance have been extensively reported with varying nomenclature and anatomical descriptions. It has been described as the mid third lateral capsular ligament (MTLCL) [2, 3] anterior oblique band of the fibular collateral ligament (AOB-FCL) [4], the capsulo-osseous layer of the ITB hypothesized to function as an anterolateral knee ligament [5, 6] the short external lateral ligament [7] and more recently the anterolateral ligament (ALL). This band, more recently termed the ALL, has been consistently described anatomically as an oblique capsular ligament coursing anteroinferiorly from the region of the lateral femoral epicondyle to the lateral tibial rim and inserting half way between Gerdy’s tubercle and the proximal fibular head [8–12]. Most recently, in a descriptive laboratory study, the anterolateral ligament described in these recent studies was not discretely identified and is thought to represent the capsulo-osseous layer of the ITB, in line with the original descriptions [13]. The band described in these studies represents the same structure and for the purposes of this study we will refer to it as the capsulo-osseous layer of the iliotibial band (CITB).

The distal insertion of the CITB has been consistently identified on MRI as inserting on to the lateral tibial rim half way between Gerdy’s tubercle and the proximal fibular head [14–18]. There have, however, been variable anatomical and MRI descriptions of the proximal origin of the CITB in relation to the lateral femoral epicondyle, FCL, ITB, and adjacent capsule [9, 10, 13, 16, 18, 19]. In the case of the MRI studies, this may be understandable, given the very close proximity of these structures and potential variability in the proximal origin.

Despite modern techniques of intra-articular ACL reconstruction, persistent pivot-shift and ALRI remains an important pattern of ongoing instability in a significant proportion of cases [20].

Recent biomechanical evidence suggests that structures of the anterolateral knee, including the CITB and more recently the distal femoral insertions of the ITB, may be important restraints in ALRI in the setting of ACL injury [21–24]. The CITB has been varyingly reported to have a role in reducing ALRI and controlling anterolateral laxity [15, 22–24].

There is debate as to the role of the CITB in resisting pivot shift in the ACL deficient knee, with some authors [15] advocating reconstruction of this structure in the pivot injured, ACL-deficient knee. Subsequent work by Kittl et al. [23, 24] suggests that the deep femoral attachments of the ITB also provides resistance to internal rotation of the tibia in near extension, such as that which occurs in the pivot-shift phenomenon. This has therefore raised the potential importance of the deep distal femoral insertions of the ITB as an additional restraint on ALRI.

A consistent distal femoral anchor of the ITB has been described as strong fibrous bands obliquely orientated from the ITB to the lateral femur just proximal to the lateral femoral condyle. These were first described by Kaplan [25] as a ‘septal insertion onto the lateral femoral condyle’ and subsequently described as an epicondylar band evident in both cadaveric dissection and on MR [26], and later by Kittl [23] as a supracondylar insertion. Most recently, this has been described as accessory insertions from the ITB inserting proximal and anterior to the femoral epicondyle [13]. In order to simplify the nomenclature, we will refer to this band as the ‘epicondylar band’. A further more proximal femoral attachment was described by Kaplan [25] as the ‘intermuscular septum inserting onto the lateral edge of the linea aspera’; this has also been described as the proximal femoral attachment [23]. In the most recent descriptive study, it has been described as the Kaplan fiber insertion onto the lateral femoral diaphysis [13]. This we have termed the ‘proximal band’ for the purposes of this paper.

Vieira et al. [6] performed an anatomic study of the ITB demonstrating three layers of the distal insertion that have already been described. A superficial layer including the ITB insertion at Gerdy’s tubercle and superficial oblique retinaculum to the patella. A deep layer including a broad insertion onto the lateral femoral diaphysis at the lateral linea aspera (termed ‘proximal band’ in this paper) and a strong ligamentous insertion towards the lateral epicondyle analogous to Fariclough’s [26] findings and termed ‘epicondylar band’ in this paper. Finally, a capsulo-osseous layer, which is described as a well-defined ligamentous structure inserting laterally to Gerdy’s tubercle, which we also term in this paper the CITB [5, 6]. This description of the anterolateral complex has again been confirmed in a recent descriptive anatomical study [13].

**Fig. 2:**
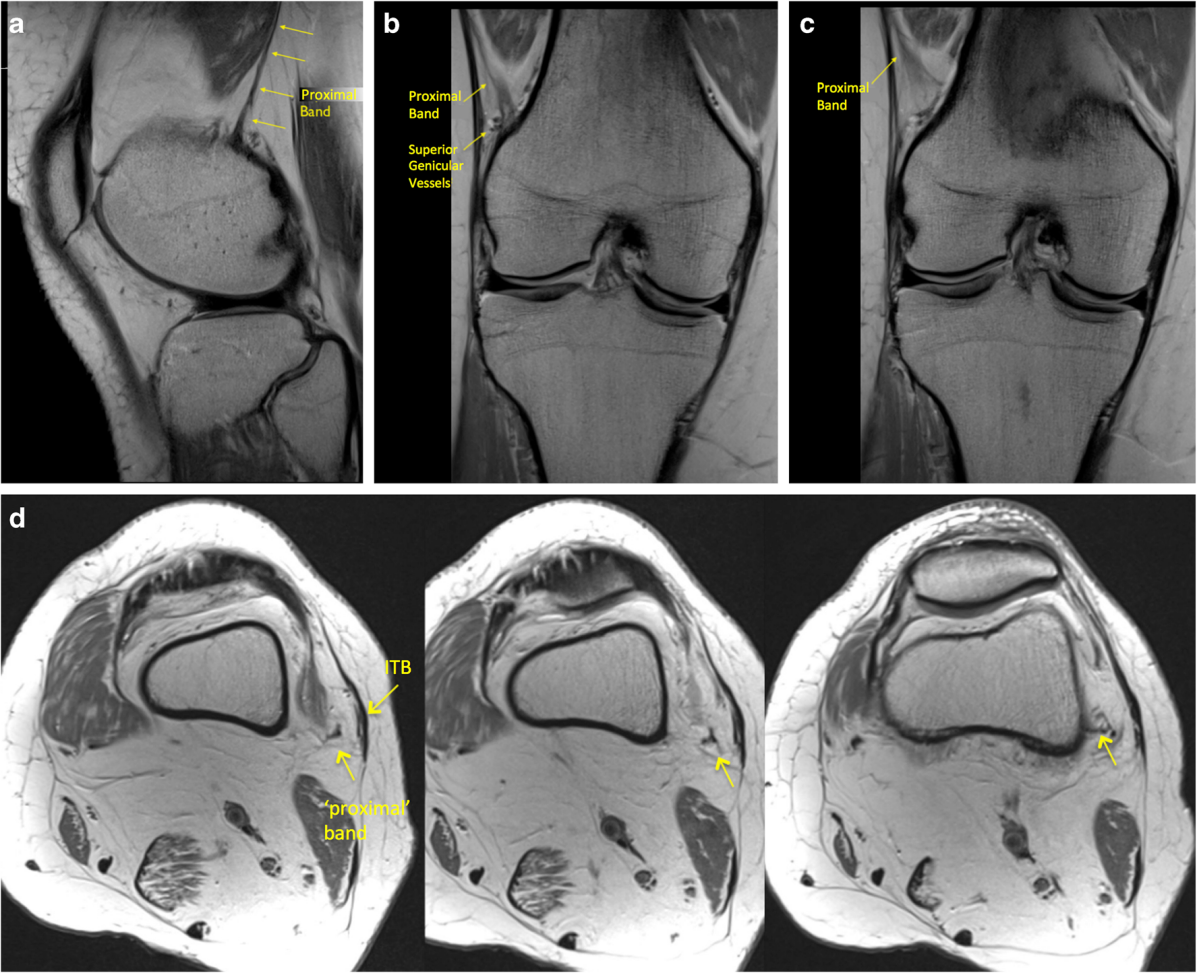
**a** Sagittal, **b**, **c** coronal, and **d**–**f** axial PD imaging demonstrating a slender low signal ‘proximal’ band of the ITB passing posterior to the vastus lateralis and attaching to the posterolateral femur (*black arrow*)

**Fig. 3:**
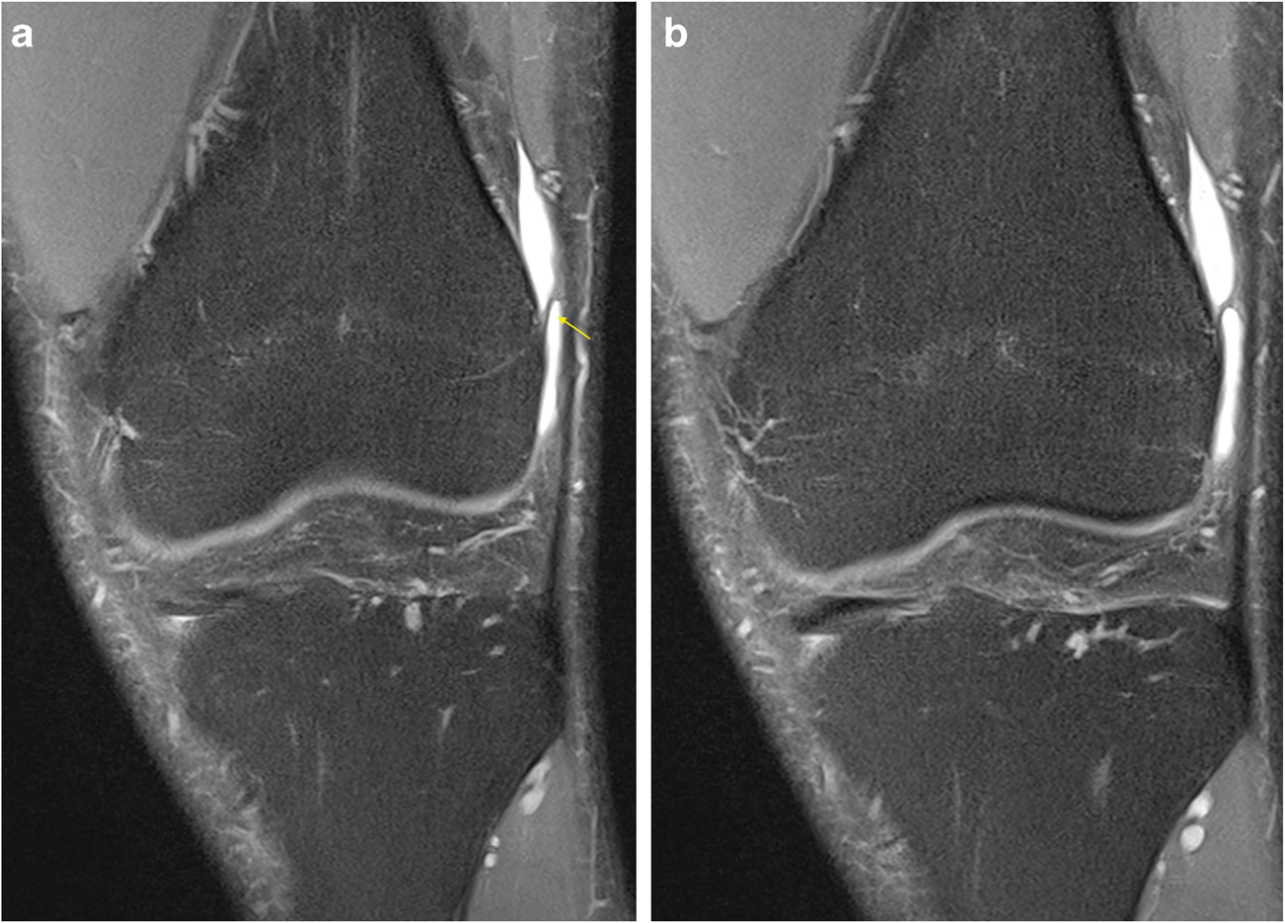
**a**, **b** Coronal PD FS imaging demonstrating a normal ‘epicondylar’ band extending from the ITB to the femoral condyle

The ‘proximal’ and ‘epicondylar’ femoral attachments of the ITB were consistently identified in our study, their anatomy and appearances on MRI are described in Figs. 2 and 3.

The importance of these additional anterolateral restraining structures and their potential impact on surgical management makes preoperative MRI evaluation useful.

A number of studies have been published describing the MRI appearances of the CITB in both normal and ACL injured knees [ 10, 14, 16, 17, 19, 27]. There have also been MRI studies published before the current interest in the CITB [27]. However, previous studies have been deficient in their description of the anatomy of the whole envelope of anterolateral structures, (‘anterolateral complex’) focusing only on the CITB and not considering the deep distal femoral insertions of the ITB. With recent biomechanical evidence suggesting that the CITB is only one part of the anterolateral restraint on pivot shift, careful evaluation of the deep-distal femoral insertions of the ITB on MRI may prove to be useful.

In this investigation, we provide the results of a retrospective analysis of the MRI appearances of the CITB and the ‘proximal’ and ‘epicondylar’ femoral insertions of the ITB in patients with a ‘normal’ knee (non ACL injured and no pivot-shift BME pattern) and compare with patients with evidence of pivot-shift BME ACL injured knees. For the first time to our knowledge, we describe the anatomy of the deep-distal femoral attachments of the ITB as identified on MRI.

## Materials and methods

We conducted a retrospective cross-sectional study of two groups of patients. These patients were identified from a list of consecutive MRI scans performed on a single Siemens 3-T MRI scanner in a hospital with a specialist knee unit, from December 2012 to November 2013. The sequences and parameters are shown in Table 1. Group 1 consisted of 20 consecutive patients with a variety of knee symptoms, whose MRI scans showed no evidence of pivot-shift BME pattern. Group 2 consisted of 20 consecutive patients identified as having a pivot-shift BME pattern with ACL injury. We excluded individuals who had undergone previous knee surgery, anyone who had suffered a direct impact injury to the anterolateral or lateral aspect of the knee, bilateral knees, and pediatric patients.

**Table 1:** MR knees reviewed and performed on a Siemens 3-T Skyra scanner with the following sequences and parameters

Sequence	PDFS TRA	PD TRA	PDFS COR	PD COR	PDFS SAG	PD SAG
TR	2430	3000	3220	3000	3530	3080
TE	34	28	28	29	38	29
Slices	30/3 mm	30/3 mm	25/3 mm	25/3 mm	27/3 mm	27/3 mm
Gap	0.6 mm	0.6 mm	0.3 mm	0.3 mm	0.6 mm	0.6 mm
Averages	1	1	2	1	2	1
Matrix	461 × 512	461 × 512	461 × 512	518 × 576	461 × 512	518 × 576
FOV	160	160	160	160	160	160
Voxel size	0.3 × 0.3 × 3.0	0.3 × 0.3 × 3.0	0.3 × 0.3 × 3.0	0.3 × 0.3 × 3.0	0.3 × 0.3 × 3.0	0.3 × 0.3 × 3.0

Proton density (PD) and proton density fat-saturated (PD FS) MR imaging was performed through the knee in three orthogonal planes. The field of view was 160 mm with a slice thickness of 3 mm and a 0.6-mm gap. Voxel size was 0.3 × 0.3 × 0.3 mm. The TE varied from between 28 and 34 and TR from 2430 to 3530. The matrix size was 461 × 512 mm, except for the PD coronal and PD sagittal imaging when it was 518 × 576 mm

Two specialist consultant musculoskeletal radiologists (15 years and 9 years experience, respectively) interpreted all MRIs by consensus. A standard data retrieval sheet was used.

### Assessment of anatomical structures

The proximal and distal attachments of the CITB, ‘proximal’ and ‘epicondylar’ distal femoral attachments of the ITB were assessed on all MRIs in order to evaluate the visibility, anatomy, and injury of these attachments.

According to recognized criteria, partial rupture was defined by altered signal within the ligament periligamentous edema and disruption of the fibers, however with no complete tear.

## Results

Demographics of both groups are displayed in table 2.

**Table 2:** Demographic results

	‘Normal’ No pivot-shift BME pattern (*n* = 20)	Pivot-shift BME pattern (*n* = 20)
Sex	11 male, 9 female	10 male, 10 female
Age range	21–67 years, median 37.5 years	18–54 years, median 35.5 years
Side	11 right, 9 left	9 right, 11 left

### CITB MRI anatomical assessment

**Fig. 4:**
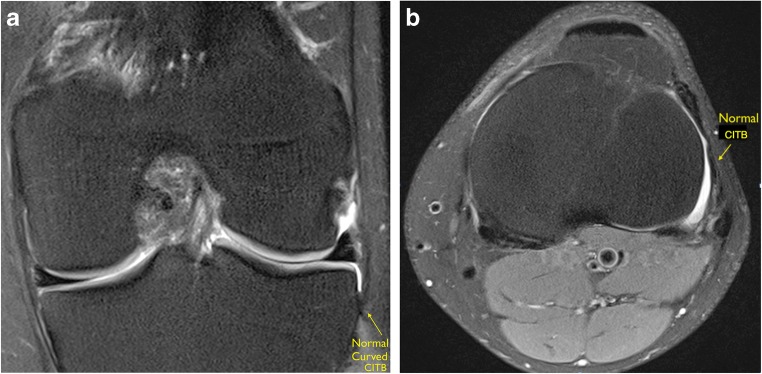
**a** Coronal PD FS and **b** axial PD FS images demonstrating a normal low signal CITB

The distal CITB was identified as a discrete ligamentous band inserting consistently immediately posterior to Gerdy’s tubercle, directly posterior to the ITB insertion (Fig. 4a, b). The proximal CITB was less distinct and had a variable proximal attachment. The results are represented in Table 3.

**Table 3:** MRI of the anatomical assessment of the CITB

	‘Normal’ No pivot-shift BME pattern (*n* = 20)	Pivot-shift BME pattern (*n* = 20)
ACL	No ACL ruptures	18 complete tear 2 high-grade, near complete
Anatomy	18/20 identified	20/20 identified
• Distal insertion	Posterior to ITB 20/20 • 16 curved insertion • 4 straight insertion	Posterior to the ITB 20/20
• Proximal insertion	15 blended with the FCL 3 unclear	12 blended with the FCL 3 blend with the fascia of the ITB 5 cases not clearly identified

### CITB MRI injury assessment

#### Group 1

In the non-pivot-shift BME group, 12 were normal at the distal attachment, five showed some altered signal with thickening, and one showed thickening with underlying bone marrow edema.

#### Group 2

**Fig. 5:**
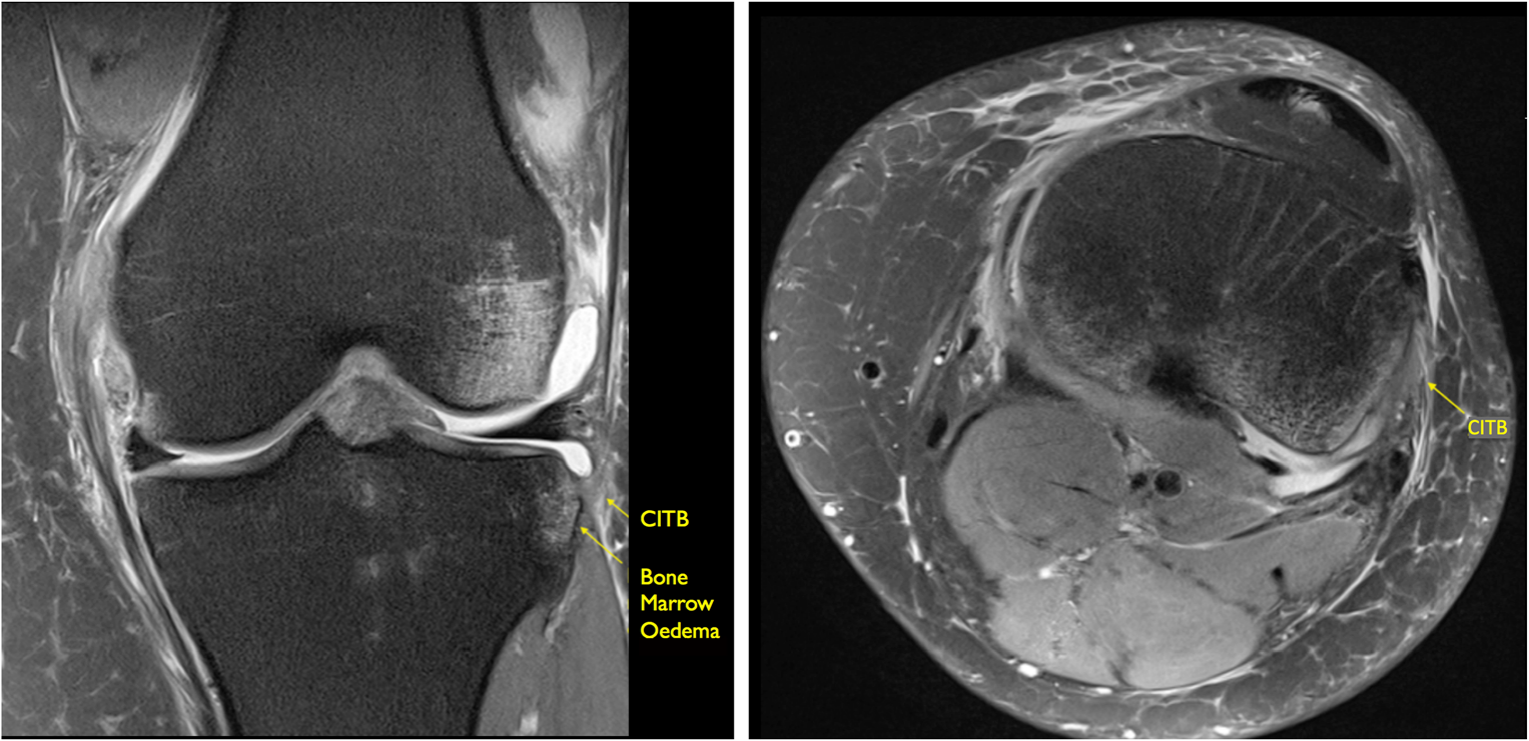
**a** Coronal and **b** axial PD FS images demonstrating increased intrinsic high signal within the CITB with increased BME at its insertion at the expected site of a Segond fracture, consistent with a partial injury

**Fig. 6:**
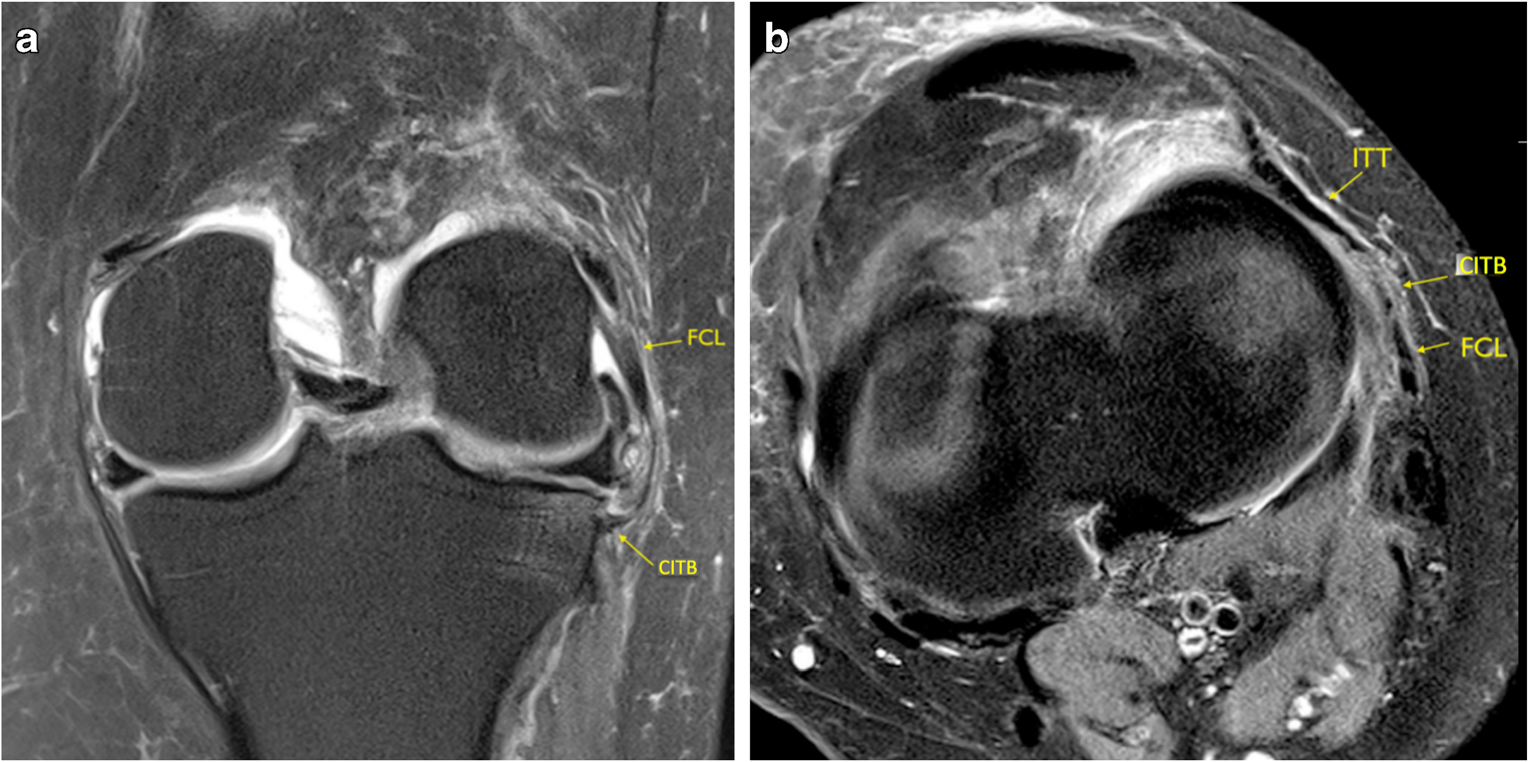
**a** Coronal and **b** axial PD FS images demonstrating the close relationship of the CITB to the FCL on coronal imaging and close proximity of the ITT, CITB, and FCL on axial imaging. There is increased intrinsic high signal within the CITB at its insertion onto the tibia

In the pivot-shift BME group, a Segond fracture was not visible in any of the 20 pivot-shift cases, although in five cases edema was identified within the lateral tibial rim at the site of the insertion of the CITB, at the expected site of a Segond fracture. All of these five cases demonstrated abnormal signal within the attaching CITB (Fig. 5a-b). Injury of the CITB and its close relationship to the ITB and FCL is demonstrated on Fig. 6a, b.

There was intrinsic high signal in the distal insertion of the CITB in 17 of the cases and three appeared normal. The results are represented in Table 4.

**Table 4:** MRI injury assessment CITB

	‘Normal’ No pivot-shift BME pattern (*n* = 20)	Pivot-shift BME pattern (*n* = 20)
ACL	No ACL ruptures	18 complete tear 2 high-grade, near complete
Anatomy	18/20 identified	20/20 identified
Distal insertion	12/18 (67%) normal appearance 5/18 (28%) increased signal and thickening 1 thickening and BME at insertion	3/20 normal appearances 17/20 (85%) increased signal and thickening • No Segond fracture • 5 cases BME at CITB insertion site

No injury of proximal attachment in either group

### Distal femoral insertions of the iliotibial band MRI anatomical assessment

Two femoral attachments of the ITB were consistently seen on MRI. One was superior to the superior lateral geniculate vessels and had an oblique course from the region of the ITB coursing inferiorly and posteriorly, passing posterior to the vastus lateralis and attaching to the linea aspera of the femur. This we termed the ‘proximal’ band Fig. 2a–f. The proximal attachment of this however could not always be identified on routine MRI knees as the origin of this band was at the proximal margin of the scan.

The second more inferior band is a curved structure in the lateral recess attaching proximal to the lateral femoral condyle; this we termed the ‘epicondylar’ band. This structure was more easily appreciated in the presence of an effusion in the lateral recess in both groups of patients Fig. 3a, b. The results are presented in Table 5.

**Table 5:** MRI anatomical assessment ‘femoral’ attachments of the ITB

	‘Normal’ No pivot-shift BME pattern (*n* = 20)	Pivot-shift BME pattern (*n* = 20)
ACL	No ACL ruptures	18 complete tear 2 high-grade, near complete
Anatomy		
Proximal band	17/20 identified	17/20 identified
Epicondylar band	13/20 identified	17/20 identified

### Distal femoral insertions of the iliotibial band MRI injury assessment

#### Group 1

In the non-pivot-shift injury knee group, the proximal femoral attachment could be seen in 17 out of 20 knees with evidence of injury in one of the 17 bands. The one with injury was in the context of vastus lateralis injury.

**Fig. 7:**
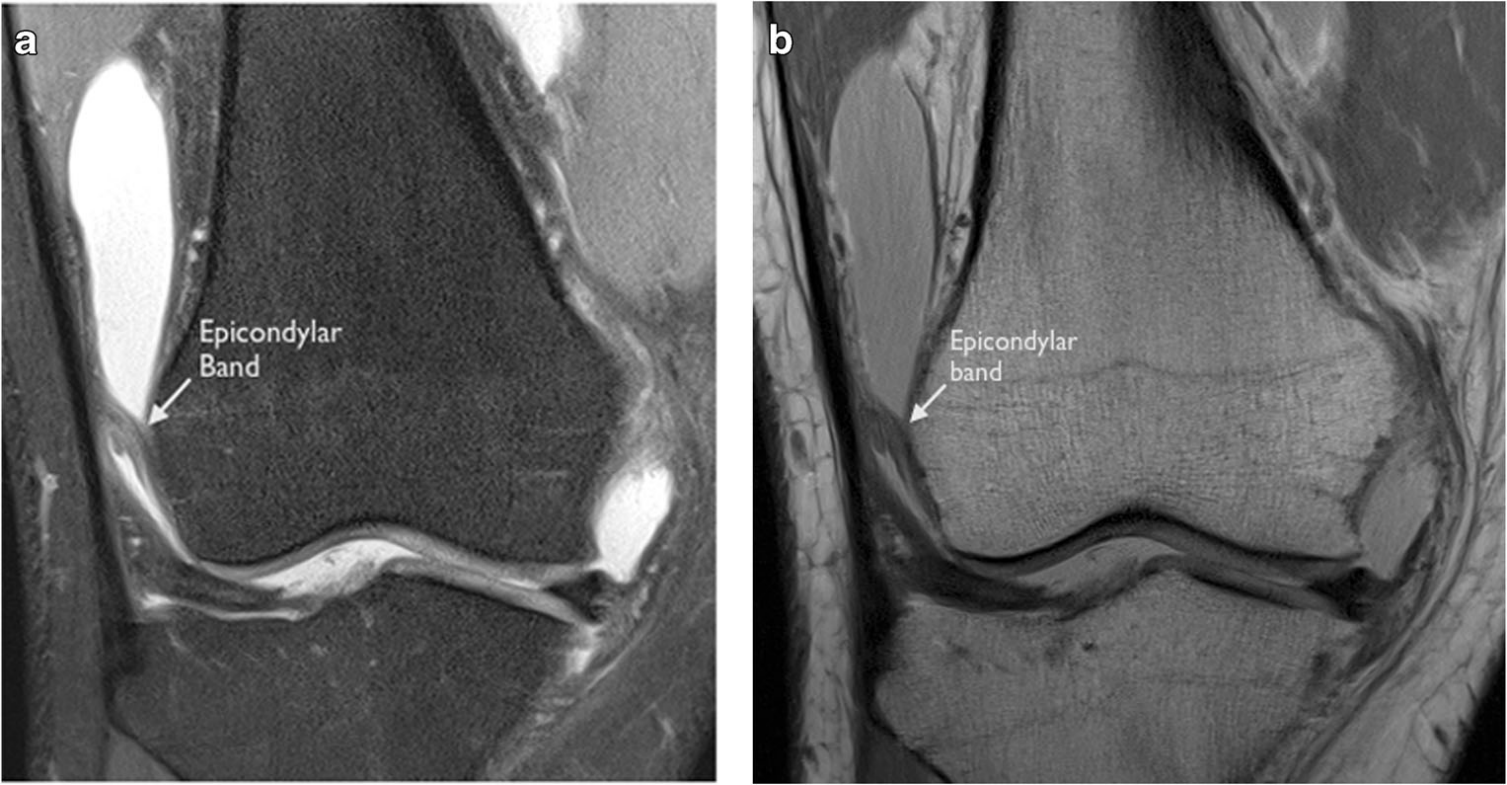
**a** Coronal PD FS and **b** coronal PD images of an epicondylar band in a non-pivot-shift knee. The band is thickened extending from the ITB to the lateral femoral epicondyle. It is more clearly visualized because of the associated effusion

The epicondylar band was visible in 13 out of 20 with evidence of injury in three of the 13 epicondylar bands (Fig. 7a, b). One of these was in the context of direct vastus lateralis muscle injury. In the other two cases, the proximal band was normal.

#### Group 2

**Fig. 8:**
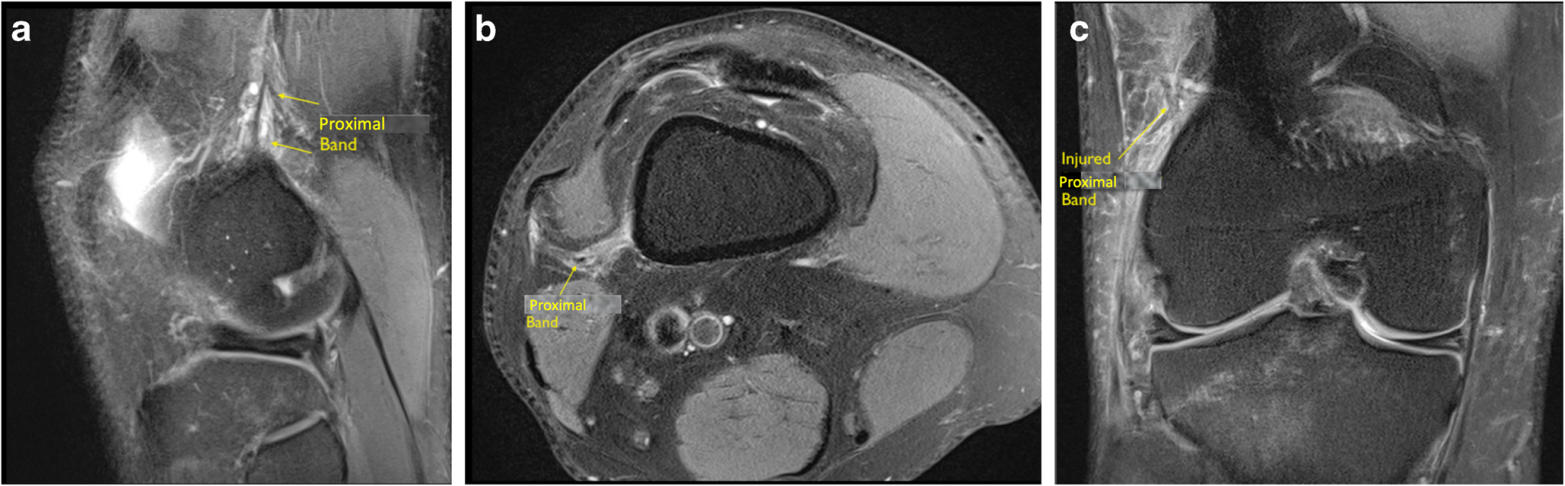
**a** Sagittal, **b** axial, and **c** coronal PD FS imaging demonstrating localized edema surrounding the proximal band of the ITB consistent with a partial injury in a pivot-shift knee

The proximal femoral attachment of the ITB was seen in 17 out of 20 knees in the ACL pivot-shift injured knee group. Fourteen of the 17 demonstrated injury (82%); all 14 showed localized edema surrounding the proximal band but four also showed diffuse lateral fat pad edema. In three cases, the proximal band was normal. In three cases, the proximal band was not clearly visible, but there was lateral fat pad edema in two of these cases (Fig. 8a–c). Results are represented in Table 6.

**Table 6:** MRI injury assessment femoral attachments of ITB

	‘Normal’ No pivot-shift BME pattern (*n* = 20)	Pivot-shift BME pattern (*n* = 20)
ACL	No ACL ruptures	18 Complete tear 2 High-grade, near complete
Anatomy		20/20 identified
• Proximal band	1/17 (0.06%) injured • 1 in context of vastus lateralis injury	14/17 (82%) injured • 14 localized edema, 4 also diffuse lateral fat edema
• Epicondylar band	3/13 (23%) injured • 1 in context of vastus lateralis injury	5/17 (29%) injured • Intrinsic altered signal and thickening

**Fig. 9:**
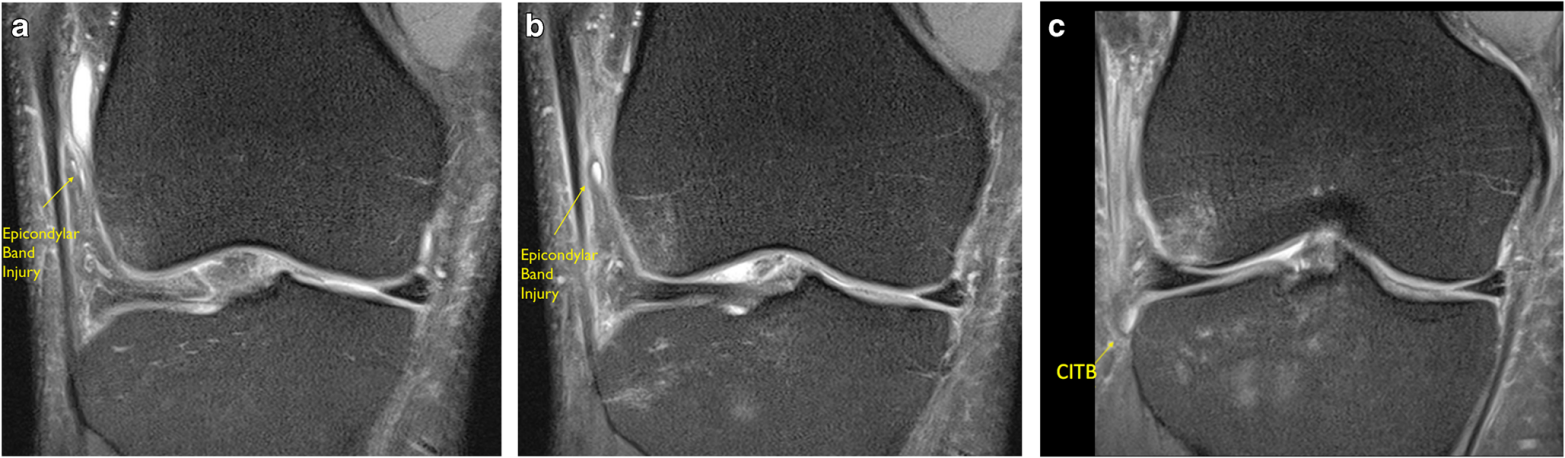
**a**, **b** Coronal PD FS imaging demonstrating partial tear of the EB in a pivot-shift injured knee with increased edema also surrounding the ITB close to its insertion. **c** Coronal PD FS imaging in the same patient demonstrates increased intrinsic high signal within the CITB consistent with a partial tear

The epicondylar band was visible in 17 cases, but could not be defined in three cases. In five of the 17 visible epicondylar bands, there was intrinsic altered signal and thickening consistent with injury Fig. 9a–c.

**Fig. 10:**
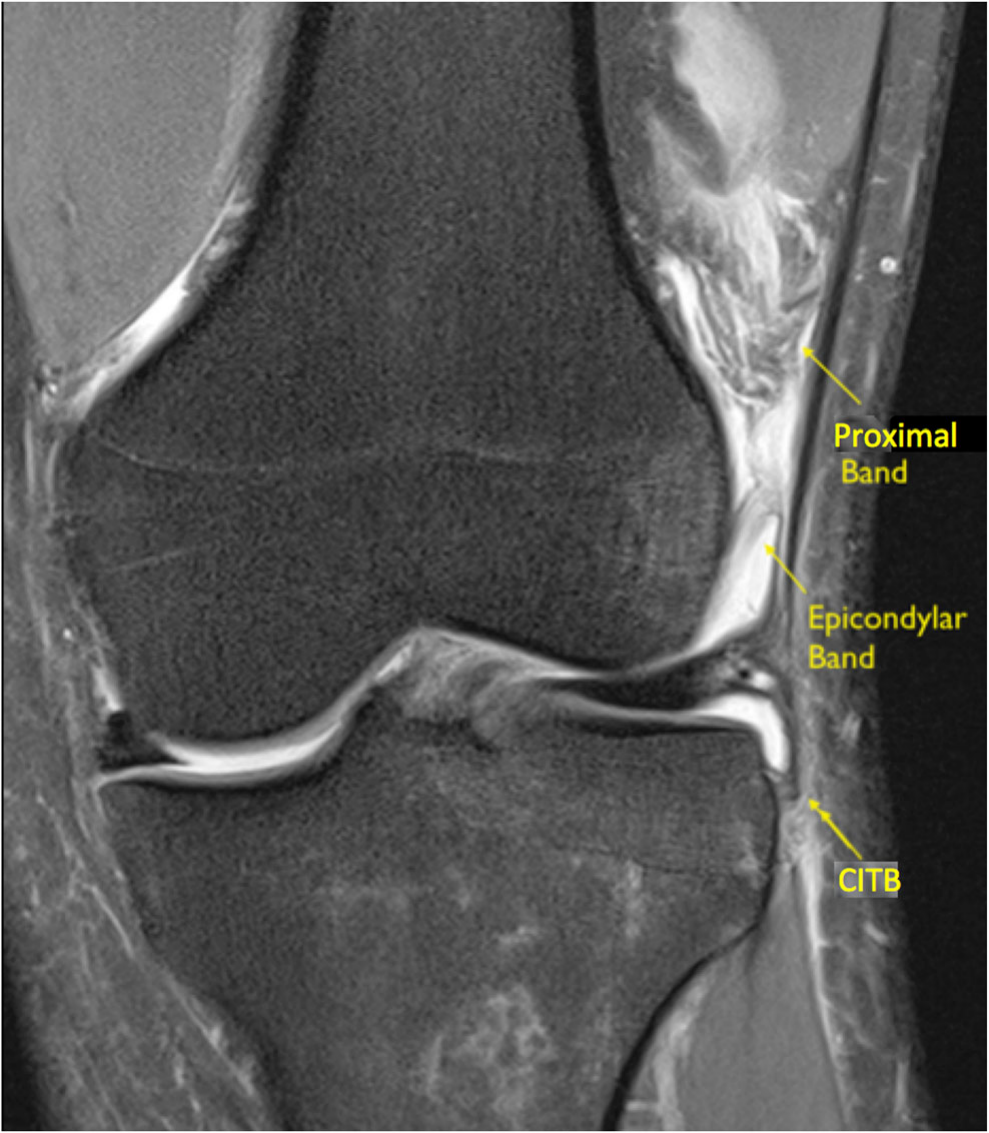
Coronal PD FS image demonstrating increased intrinsic high signal within the epicondylar and proximal bands of the ITB as well as the CITB consistent with a partial injury in all three structures in a pivot-shift knee

Of the five in the pivot-shift group, four had associated injury in the region of the proximal band Fig. 10. Results are represented in Table 5.

### Tibial insertion of the iliotibial band

The distal ITB showed subtle abnormal increased intrinsic high signal in three of the “normal” non-pivot-shift knees.

No abnormality of the distal ITB insertion was seen in the ACL pivot-shift injury group.

An online statistical package was used to calculate the sensitivity, specificity and subsequently the PPV and NPV for injury of the Anterolateral Structures of the knee in pivot-shift BME pattern ACL injured knees, which is presented in Table 7.

**Table 7:** PPV and NPV for injury of the anterolateral structures of the knee in pivot-shift BME pattern ACL injured knees

	PPV	NPV
CITB 17/20 (85% injured)	74% (95% CI is 52–90%)	80% (95% CI 52–96%)
Proximal femoral band ITB 14/17 (82% injured)	93% (95% CI is 68–100%)	84% (95% CI is 60–97%)
Epicondylar band ITB 5 /17 (29% injured)	62% (95% CI is 24–91%)	45% (95% CI is 24–68%)

## Discussion

Our study aimed to describe the MRI anatomy of the anterolateral structures of the knee and the presence of injury to these structures in knees with a pivot-shift BME pattern indicative of ACL injury and compare this with non-pivot-shift non-ACL injured knees.

Although studies have been published that review the MRI appearance of the CITB, there has been no published work, to our knowledge, evaluating the MRI appearance of the distal ITB femoral attachments.

The CITB has been extensively investigated both anatomically and on MRI [8–18]. Our study consistently demonstrated the distal insertion of the CITB at the anterolateral tibial rim at the posterior margin of the superficial ITB insertion. This concurs with multiple previous anatomical [6, 8–12] and MRI [14, 16–19] studies. We found that the proximal origin of the CITB was more difficult to define separately. No clear separate femoral insertion was identified on any of the examined MRI studies. This concurs with the findings of previous anatomical and MRI investigations where there have been variable reports of the origin of this ligament [9, 10, 16, 18, 19]. Some studies have stated that the proximal CITB blends with the FCL close to its femoral origin [10, 28]. Our study demonstrated a high rate of apparent blending of the CITB with the anterior margin of the FCL analogous to the anterior oblique ligament [4]. The close apposition of these structures anatomically may explain the lack of visible separation of these structures, even on high-resolution MRI imaging.

The distal tibial insertion of the CITB was the only injured component of the CITB in our study, the PPV of CITB injury for pivot-shift injury was 74% and NPV 80%. Proximal CITB injury was not identified. This differs from Helito’s study [29] where approximately a third demonstrated a CITB injury and the majority were proximal.

Interestingly, in our studied group, there was no discrete Segond fracture, but five cases demonstrated prominent bone marrow edema at the CITB insertion at the lateral tibial rim at the expected site of a Segond fracture.

The distal femoral attachments of the ITB were assessed. The deep femoral attachments of the ITB have been described by Kaplan [25] in 1958, Fairclough [26], Vieira [6], and Kittl [23]. A deep attachment of the ITB towards the lateral femoral epicondylar region, which we have termed the epi*condylar band*, was identified as a curved low signal band which runs from the distal ITB through the lateral recess and onto the lateral femoral epicondyle. This band was identified on MR in 65% of the non-pivot-shift group and 85% of the pivot-shift group. This disparity in identification between the two groups we attributed to the presence of an effusion, more commonly present in the pivot-shift group, which distended the lateral recess allowing increased visualization of this band due to an arthrographic effect. This phenomenon was also described in Fariclough’s study [26].

Abnormalities of this epicondylar band showed a PPV of (62%) and NPV (45%) for pivot-shift injury. Kaplan and Vieira’s dissection of the ITB clearly describes a second femoral attachment to the femoral diaphysis laterally at the linea aspera [6]. More recently, Kittl et al. have described a proximal attachment of the ITB to the femur [23]. This we have termed the ‘proximal’ band. This was consistently identified in our study as a diagonal, slender, low signal band passing posteroinferiorly from the ITB, coursing posterior to the vastus lateralis obliquus muscle and attaching to the posterolateral margin of the femur at the linea aspera above the superolateral genicular vessels. Routine MRI scans did not always cover the proximal attachment of the proximal band to the ITB and more proximal scanning may be required on future routine MRI in order to assess the proximal extent of this band.

Our study demonstrated this band in 85% of both the pivot-shift and non pivot-shift groups. Abnormalities of the proximal band showed a high PPV of 93% and NPV 84% for pivot-shift injury.

Interestingly, the distal tibial Gerdy’s tubercle attachment of the ITB was normal in all cases in the pivot-shift group and mildly abnormal in three of the non-pivot-shift group. Hence, in our study, the tibial insertion of the ITB was not injured in the pivot-shift phenomenon. This is in contrast to Flores et al. who do report the posterior fibers of the distal tibial ITB occasionally being attached to the Segond fracture fragment.

The current study shows that both the CITB and proximal femoral attachments of the ITB are commonly injured during pivot-shift injury. They are therefore good markers for ACL injury even in the absence of a Segond fracture and should be evaluated on all MRIs as they may prove important in the further management of ALRI.

The question remains: how important are these components of the anterolateral envelope in restraining pivot-shift phenomenon and ALRI post pivot-shift injury/ACL injury and post-ACL surgery?

Biomechanical studies have suggested that the CITB is a consistent structure that contributes to tibial internal rotation resistance [15, 22, 30]. In addition, Monaco et al. [21], utilizing a navigation system, demonstrated the clinical impact of anterolateral reconstruction in the context of ACL repair and demonstrated that this reduced ALRI [21]. Recently, a further study showed that while the CITB does provide some resistance, the femoral attachments of the distal ITB may be stronger and better aligned to provide this action [24, 31].

Our study has described the MRI appearance of the ITB femoral attachments for the first time. The MRI appearances have been assessed in both pivot-shift BME pattern with ACL injury and non-pivot-shift BME knees without ACL injury. This has allowed us to confirm that these structures may be routinely identified and assessed for damage resulting from pivot-shift injury. Our results support the laboratory biomechanical work suggesting that the ITB femoral distal attachments may play an important role in anterolateral rotational stability [32–34].

Since these anterolateral structures are injured during a pivot-shift phenomenon and have been shown to be biomechanically important in resisting pivot shift, it has been suggested that reconstructing some aspect of these structures as an adjunct to intra-articular ACL reconstruction may reduce the incidence of post-operative pivot-shift ALRI [9, 14, 23, 24, 31]. As a result, lateral extra-articular procedures concomitant with intra-articular ACL reconstruction have become increasingly popular.

However, simply seeing damage to these anterolateral structures, as seen in this study, does not necessarily indicate anterolateral reconstruction is required as being extra-articular they may well heal. The question remains as to which of the anterolateral structures, CITB, proximal, or epicondylar femoral bands of the ITB need to be addressed surgically, if any at all.

The CITB appeared curved at its distal tibial insertion in the majority of the knees in our study, which suggests that it may be slack in a small degree of flexion as is the case during a knee MRI. This would mean it would be less effective at controlling anterolateral tibial rotation close to extension. The pivot-shift phenomenon tends to occur around 20–30 degrees of flexion and so the CITB would need to tighten considerably to be effective in preventing ‘pivoting’. Hence, its major role may be in preventing rotation at greater angles of flexion [22] but that would question any need to replicate it in ACL reconstruction surgery. It may be that the most important initial restraint to tibial rotation in lesser degrees of flexion is the distal femoral attachments of the ITB. Only once the major restraint has failed will lesser structures be loaded, with, for example, the CITB being injured in more extreme injury.

Our work is important as, for the first time, the femoral attachments of the ITB have been identified and described anatomically on MRI. These bands are consistently seen and may be evaluated for injury.

This is clinically important given the resurgence of interest in anterolateral reconstruction in the context of ACL repair. Hence, preoperative MRI evaluation of these structures may play a critical role in surgical planning. Lateral tenodesis, although non-anatomical, has been an effective surgical intervention in controlling tibial internal rotation.

## Further work

Our study has suggested that the distal femoral ITB attachments, which we have designated the ‘proximal’ and ‘epicondylar’ bands, are commonly injured in pivot-shift injury. This correlates with recent biomechanical studies describing it as an important structure in the resistance of ALRI. Future work comparing non-pivot-shift BME ACL injured knees with pivot-shift BME injured knees are indicated in order to support the biomechanical assumption that the anterolateral structures are a restraint.

This will help inform appropriate surgical management in the context of the acutely injured knee.

## Limitations

This study was retrospective and contained only a small number of subjects, although this was enough to show significant positive predictive values for both CITB and deep femoral ITB injuries in ACL injured knees. The MRIs were also evaluated by consensus and were not blinded. The control group was a sample of individuals with a variety of clinical indications for scanning. Further work to assess if this injury pattern is more common in certain sports would be helpful.

## Conclusions

Injury of the CITB, ‘proximal’, or ‘epicondylar’ deep femoral attachments of the ITB are good markers for ACL injury even in the absence of a Segond fracture. These structures are considered to be the primary soft tissue restraint to tibial internal rotation and should be evaluated on all MRIs as this may prove important in the further management of ALRI.
